# Strongly-coupled quantum critical point in an all-in-all-out antiferromagnet

**DOI:** 10.1038/s41467-018-05435-7

**Published:** 2018-07-27

**Authors:** Yishu Wang, T. F. Rosenbaum, A. Palmer, Y. Ren, J.-W. Kim, D. Mandrus, Yejun Feng

**Affiliations:** 1 Division of Physics, Mathematics, and Astronomy, California Institute of Technology, Pasadena, CA 91125 USA; 2 Department of Physics, The James Franck Institute, The University of Chicago, Chicago, IL 60637 USA; 3 The Advanced Photon Source, Argonne National Laboratory, Argonne, IL 60439 USA; 4 Department of Materials Science and Engineering, University of Tennessee, Knoxville, TN 37996 USA; 5 Materials Science and Technology Division, Oak Ridge National Laboratory, Oak Ridge, TN 37831 USA; 6 Okinawa Institute of Science and Technology Graduate University, Onna, Okinawa 904-0495 Japan

## Abstract

Dimensionality and symmetry play deterministic roles in the laws of Nature. They are important tools to characterize and understand quantum phase transitions, especially in the limit of strong correlations between spin, orbit, charge, and structural degrees of freedom. Here, using newly-developed, high-pressure resonant X-ray magnetic and charge diffraction techniques, we have discovered a quantum critical point in Cd_2_Os_2_O_7_ as the all-in-all-out antiferromagnetic order is continuously suppressed to zero temperature and, concomitantly, the cubic lattice structure continuously changes from space group *Fd-3m* to *F-43m*. Surrounded by three phases of different time reversal and spatial inversion symmetries, the quantum critical region anchors two phase lines of opposite curvature, with striking departures from a mean-field form at high pressure. As spin fluctuations, lattice breathing modes, and quasiparticle excitations interact in the quantum critical region, we argue that they present the necessary components for strongly-coupled quantum criticality in this three-dimensional compound.

## Abstract (web summary)

The critical behavior of a continuous phase transition is determined by both symmetry and dimensionality. Wang et al. present evidence that the cubic all-in-all-out spin order in Cd_2_Os_2_O_7_ leads to a realization of a non-mean-field strongly-coupled quantum phase transition in three dimensions.

## Introduction

Fundamental symmetry constraints determine allowable collective states in solids^1^. Of particular recent interest is broken inversion symmetry in non-centrosymmetric materials, which can lead to hidden topological order and odd-parity superconductivity^1–3^. With the added consideration of broken time-reversal invariance, exotic magnetic states can emerge. A quantum critical point, where quantum fluctuations tie together competing ground states, is a fertile region to investigate and manipulate intertwined charge, spin, and structural degrees of freedom under different symmetry conditions. However, many experimental systems either manifest first-order quantum phase transitions without critical behavior, as exemplified by itinerant ferromagnets^4^, or simply follow mean-field behavior^5,6^. Strongly-coupled systems with pronounced spin-orbit interactions provide a potential means to move to non-trivial criticality in three dimensions^4,5^. This scenario has been proposed for all-in-all-out (AIAO) antiferromagnetic order on a pyrochlore lattice^4^, but remains to be identified experimentally.

The AIAO arrangement of spins on the pyrochlore lattice is an unusual form of magnetism that preserves the underlying cubic lattice symmetry. With strong spin-orbit coupling and low itinerant electron density, compounds with AIAO spin order are desirable candidates to explore non-trivial quantum critical behavior in three dimensions^5^. AIAO spin order has been observed in FeF_3_, Nd_2_Zr_2_O_7_, *A*_2_Ir_2_O_7_ (*A* = Sm, Eu, and Nd), and Cd_2_Os_2_O_7_ (refs.^7^^–^^12^) and suggested for additional *A*_2_Ir_2_O_7_ systems with *A* = Y, Lu, Gd, Tb, Dy, Ho, and Yb^13^. For our purposes, Cd_2_Os_2_O_7_ is most desirable. With the transition temperature (*T*_N_ = 227 K) roughly 60% higher than all other *A*_2_Ir_2_O_7_ members, Cd_2_Os_2_O_7_ should demonstrate the strongest correlation effects. Moreover, among spin-orbit coupled 5*d* compounds, only Cd_2_Os_2_O_7_ and Nd_2_Ir_2_O_7_ consistently manifest both an antiferromagnetic insulating phase and a metallic paramagnetic phase with d*ρ*/d*T* > 0 (refs.^13^^–^^15^). It remains unclear whether iridates such as Eu_2_Ir_2_O_7_ and Sm_2_Ir_2_O_7_ are metallic or insulating in the paramagnetic phase, presumably due to vacancies and site disorder from the 3+/4+ valence condition^16^. By comparison, Cd_2_Os_2_O_7_ exhibits less site disorder from its 2+/5+ valence condition, with no extraneous spin order arising from the *A* site of the *A*_2_(Ir,Os)_2_O_7_ structure^14^. Both pressure^14,17,18^ and chemical tuning^15,18^ of the *A* site drive the insulating transition to lower temperature in the *A*_2_(Ir,Os)_2_O_7_ compounds, but little is known about the behavior of the AIAO magnetic order.

Here, to address this issue directly, we present results on the evolution of the spin, orbit, and lattice degrees of freedom, using polarization analysis of resonantly diffracted X-rays from Cd_2_Os_2_O_7_ under diamond anvil cell pressures. This resonant diffraction technique is highly challenging with regard to both maintaining the single-crystal quality at cryogenic temperatures and tens of giga-Pascal pressures, and efficiently detecting weak magnetic diffraction signals from miniature samples of 5 × 10^−5^ mm^3^ size. Nevertheless, AIAO magnetic order and 5*d* orbital order can be resolved directly and tracked to the highest pressures under resonant conditions in the polarization switching *π*−σ channel of the (6, 0, 0) and (4, 2, 0) forbidden lattice orders, respectively^9^. The lattice symmetry and space group assignments were detected via the *π–π*’ scattering channel.

## Results

### The magnetic quantum phase transition

**Fig. 1:**
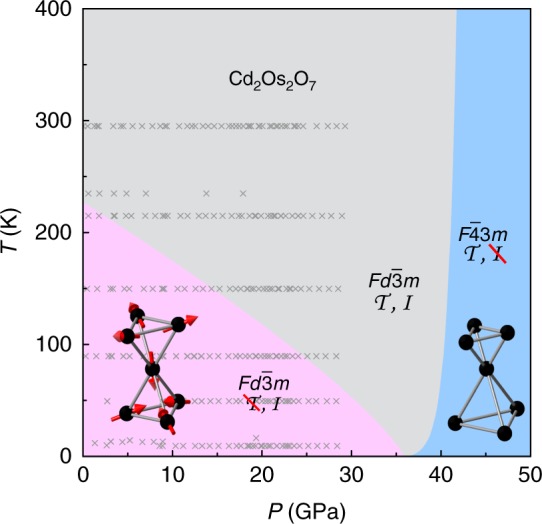
Symmetries across the *P–T* phase diagram. The Cd_2_Os_2_O_7_ lattice retains its cubic symmetry throughout the probed *P–T* phase space, but continuously transitions between *Fd-3m* and *F-43m* space groups. The *Fd-3m* lattice symmetry was verified by optical Raman scattering from 0–29 GPa and 10–300 K (gray crosses), while both phases of magnetism and structure (pink and blue shading) were inferred from X-ray diffraction measurements at *T* = 4 K. The metallic paramagnetic phase in the low-pressure *Fd-3m* space group has both spatial inversion ($\cal{I}$) and time reversal ($\cal{T}$) symmetries. For the spin degrees of freedom, time reversal symmetry is broken in the low-pressure AIAO phase. On the high-pressure side, the *F-43m* space group breaks the spatial inversion symmetry, introduces a tetrahedral breathing distortion (inset), and restores the time reversal symmetry with disordered spins. The coincidence of continuous magnetic and structural phase transitions at a single quantum critical point suggests strong coupling between spin, orbit, lattice, and (potentially) charge degrees of freedom in this *5d* pyrochlore compound

**Fig. 2:**
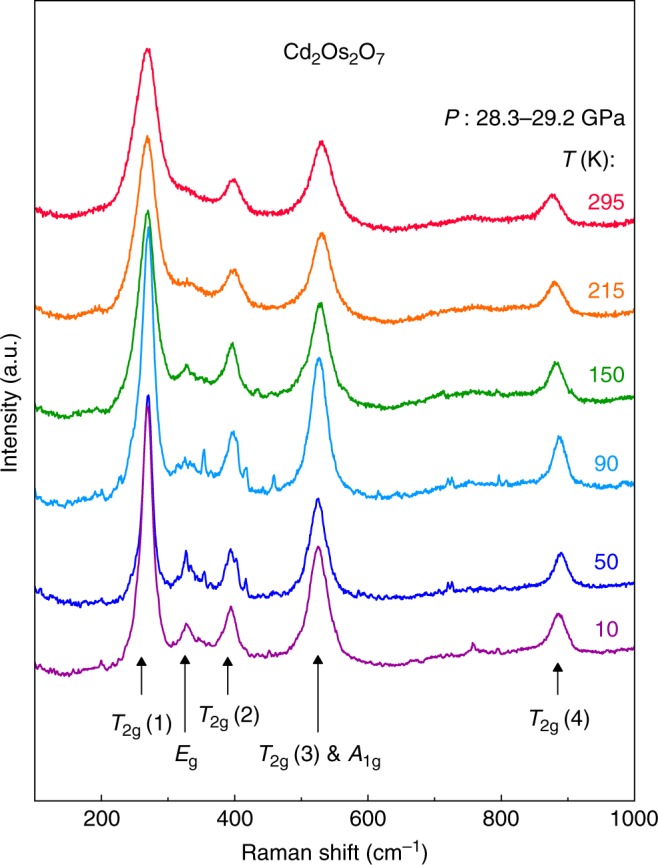
Optical Raman scattering. Raw Raman spectra of Cd_2_Os_2_O_7_ at various temperatures from 10 to 300 K and our highest measured pressure, *P* ~ 29 GPa. We only observe the six modes consistent with the *Fd-3m* space group. The *T*_2g_(3) and *A*_1g_ modes merge under their broadened peaks

**Fig. 3:**
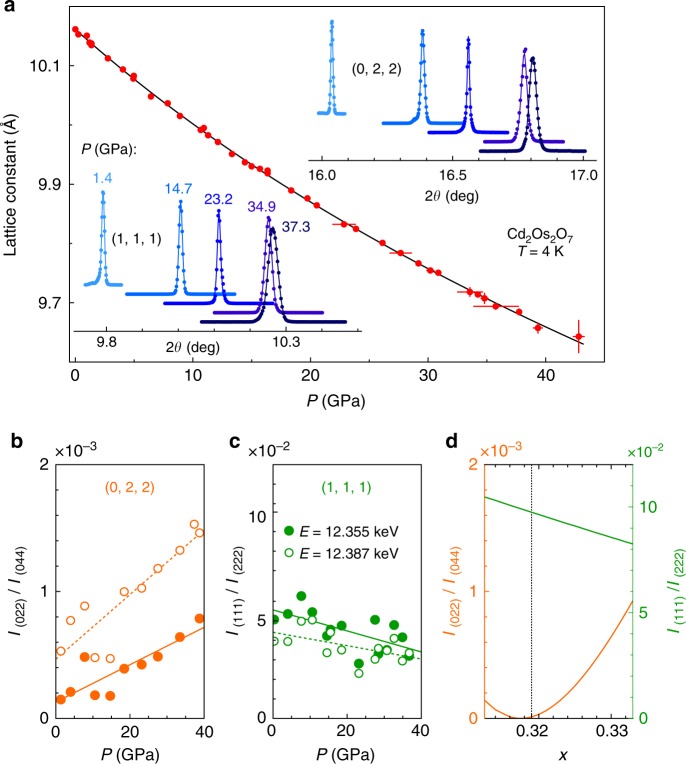
Lattice evolution under pressure. **a** Pressure evolution of the lattice constant (red solid circles) was fit to a two-parameter Birch equation of state with *B* = 190.4 ± 3.6 GPa, and *B*’ *=* 4.2 ± 0.2. Vertical error bars represent 1σ s.d. counting statistics, and horizontal error bars represent the full range of pressure inhomogeneity during a measurement. (insets) Longitudinal (*θ*/2*θ*) scans of (1, 1, 1) and (0, 2, 2) orders measured at various pressures using 12.387 keV X-rays verify the cubic symmetry. **b**–**c** Pressure evolution of integrated diffraction intensities of (0, 2, 2) and (1, 1, 1) orders, normalized by (0, 4, 4) and (2, 2, 2) orders, respectively. The measurement was performed under either resonant (*E* = 12.387 keV, solid circles) or off-resonant (*E* = 12.355 keV, open circles) conditions. These two orders are sensitive to O 48 *f* sites in the unit cell, and develop in opposite fashion up to 40 GPa. **d** Simulated (0, 2, 2) and (1, 1, 1) intensities as a function of *x*. The overall percentage changes of (0, 2, 2) and (1, 1, 1) give an *x* increasing from 0.319 at *P* = 0 (ref.^14^) to approximately 0.325 at *P*_c_

We summarize in Fig. 1 the symmetry evolution under pressure. At ambient pressure, Cd_2_Os_2_O_7_ has the pyrochlore structure of the *Fd-3m* space group (No. 227). Optical Raman scattering maps out a uniform *Fd-3m* phase up to *P* = 29 GPa and between *T* = 10 and 300 K. The phonon modes characteristic of that space group develop smoothly over the entire range (Fig. 2). The overall cubic symmetry is further verified by X-ray diffraction up to 41 GPa at 4 K (Fig. 3), as lineshapes of the (1, 1, 1), (2, 2, 2), (0, 2, 2), (0, 4, 4), and (4, 0, 0) diffraction orders remain single peak. The cubic lattice constant decreases smoothly under pressure, without discernable discontinuity at the quantum critical point *P*_c_ = 35.8 GPa (Fig. 1, discussed below). While the *Fd-3m* space group is uniquely determined by the unit cell’s lattice constant and one free coordinate *x* for oxygen position on 48*f* sites that characterizes the trigonal distortion of the OsO_6_ cluster^14,19^, diffraction intensities at (1, 1, 1) and (0, 2, 2) show a continuous evolution through the quantum phase transition, with *x* increasing by a small amount from 0.319 at *P* = 0 (ref.^14^) to ~0.325 at *P*_c_ (Fig. 3b, c). Neither the AIAO antiferromagnetic order nor the continuous space group evolution within the cubic symmetry (Fig. 1) would be detectable by macroscopic approaches such as electrical transport. Instead, we address these issues using resonant single crystal diffraction with polarization analysis at the Os *L*_2_ edge.

**Fig. 4:**
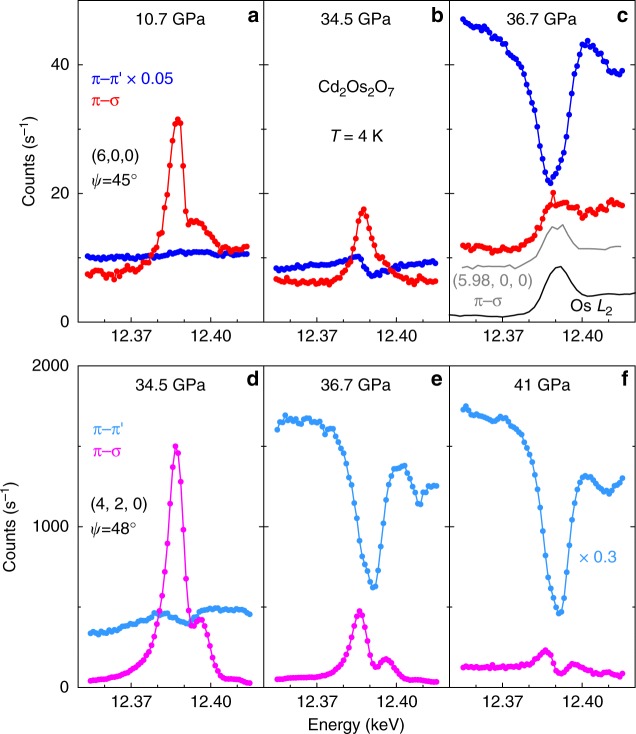
Polarization-sensitive resonant diffraction data under pressure. Raw energy scan profiles at both (**a**–**c**) (6, 0, 0) and (**d**–**f**) (4, 2, 0) orders from two separate polarization channels (*π*–σ in red/pink and *π–π*’ in navy/aqua solid circles), expressed in counts s^−1^ for 100 mA synchrotron storage ring current. The azimuthal angel *ψ* relative to the (0, 0, 1) vector is specified for each order. At (6, 0, 0), the *π*–σ channel manifests magnetic resonant spectra whose intensity decreases continuously with pressure. Beyond 36 GPa (**c**), the sharp resonance disappears, and a background similar to the Os *L*_2_ absorption edge shape remains, which comes from X-ray fluorescence that passed through the polarization analyzer. Its origin is demonstrated by a comparison with an energy scan at (5.98, 0, 0) (gray line, **c**), where no magnetic diffraction is expected, and also with an Os *L*_2_ absorption spectrum at 39.3 GPa (black line, **c**). The fluorescence part of the background intensity is dependent on the detector slit size. At (4, 2, 0), the *π*–σ channel reveals the orbital ordering via the anisotropic tensor susceptibility resonance. For both (6, 0, 0) and (4, 2, 0) orders, the π-π’ charge diffraction intensities rise dramatically at pressures above the magnetic phase boundary, with a small leakage into the π-σ channel becoming apparent in **f** through the polarization analyzer (Methods)

**Fig. 5:**
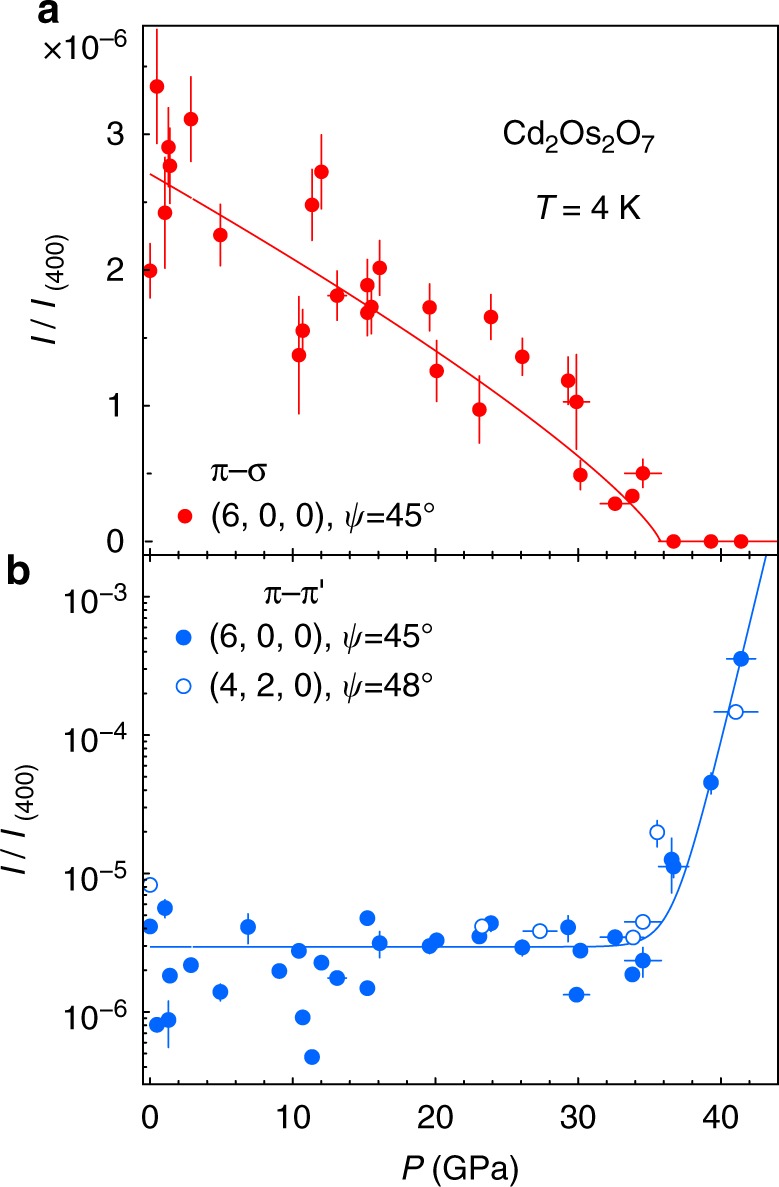
Continuous magnetic and structural quantum phase transitions. **a** Magnetic diffraction intensity was measured at (6, 0, 0) and in the *π*−σ channel, with a power law fit (solid line) to model the evolution over the whole pressure range. **b** Lattice diffraction intensities, measured at both the (6, 0, 0) and (4, 2, 0) orders in the *π–π*’ channel, indicate a continuous switching between the *Fd-3m* and *F-43m* space groups with a phase boundary that rises effectively exponentially. All intensities were measured at 4.0 ± 0.5 K with integration of sample mosaic profiles at specified azimuthal angle *ψ* using 12.387 keV X-rays. Vertical error bars represent 1σ s.d. counting statistics, and horizontal error bars represent the full range of pressure inhomogeneity during a measurement

Resonant diffraction at both (6, 0, 0) and (4, 2, 0) in the *π*−σ and *π−π*’ polarization channels are displayed in Fig. 4 for pressures that traverse the quantum phase boundary. The magnetic diffraction intensity *I*_(6, 0, 0)_ in the *π*−σ channel at *E* = 12.387 keV, which scales to the ordered staggered moment <*m*> as *I* ~ <*m*>^2^, decreases continuously with increasing pressure (Fig. 5a). The scatter in the data makes it difficult to identify definitively the quantum critical region, but a phenomenological fit of the intensity data as *I*~ <*m*>^2^~(*P*_c_–*P*)^2*β*^ over the whole pressure range gives a critical pressure *P*_c_ = 35.8 ± 0.7 GPa and an exponent *β* = 0.40 ± 0.04 for the order parameter <*m*>. From energetic considerations of localized 3*d*-5*d* spins^20,21^, our magnetic diffraction results at the low temperature limit of 4 K also provide a means to estimate the AIAO magnetic phase boundary through *T*_N_ ~ *ΔL* ~ <*m*>^2^, where *ΔL* is the external magnetostriction that develops under <*m*>. In AIAO-ordered pyrochlores where the cubic lattice symmetry is preserved by the magnetic order and the phase transition is continuous, the external magnetostriction *ΔL* is difficult to observe over the lattice’s thermal expansion. Nevertheless, a non-monotonic evolution of the lattice constant with temperature, *a*(*T*), for AIAO order at very low *T* has been demonstrated in Nd_2_Ir_2_O_7_ (*T*_N_ = 33.5 K and Δ*a*/*a* ~1 10^−4^)^22^. Following this logic, we identify the magnetic phase boundary *T*_N_(*P*) of Cd_2_Os_2_O_7_ in Fig. 1 via the relationship *T*_N_ ~ *I* ~ <*m*>^2^. At high pressure, for *P* *>* *P*_c_, the presence of a charge resonance at (4, 2, 0) verifies that the unoccupied *t*_2g_ orbitals remain the same in promoting the resonance behavior (Fig. 4e, f). At the same time, the absence of a resonance at (6, 0, 0) importantly marks the vanishing of the staggered moment <*m*> and the long-range antiferromagnetic order (Fig. 4c).

### The lattice quantum phase transition

With the disappearance of AIAO order at the magnetic critical point *P*_c_, the charge diffraction starts to gain intensity in the *π–π*’ channel for both (6, 0, 0) and (4, 2, 0) (Fig. 4), and grows continuously for *P* *>* *P*_c_ in an effectively exponential manner (Fig. 5b). We emphasize that these are forbidden diffraction orders in the *Fd-3m* space group. The energy dependence manifests no resonance, but is instead consistent with the inverted shape of the Os *L*_2_ absorption edge. At 41 GPa, 15% above *P*_c_, there is no diffraction intensity at forbidden orders (3, 0, 0), (5, 0, 0), and (2, 1, 1). With preserved cubic symmetry, *F-43m* is the only maximal non-isomorphic subgroup of the original *Fd-3m* space group that is consistent with the measured diffraction results. We note that the pressure-induced AIAO quantum phase transition in Cd_2_Os_2_O_7_ differs from the chemical tuning scenario of the *A*_2_Ir_2_O_7_ series (*A* from Sm to Pr), where the lattice inversion symmetry is preserved throughout^13^.

The space group *F-43m* breaks lattice inversion symmetry, opening possibilities for a number of exotic states. We did not detect further symmetry breaking such as the small tetragonal distortion in Cd_2_Re_2_O_7_ that would remove the three-fold rotational symmetry^23^. The broken inversion symmetry derives from differently sized adjacent tetrahedra of the Os and Cd sublattices, which are no longer centrosymmetric and can be regarded as fully-softened breathing modes^3^ with spontaneous symmetry breaking. These breathing modes disappear above *T*_c_ ~ *K*|Δ_Os_|^2^, with the lower *F-43m* symmetry replaced by the higher *Fd-3m* symmetry with adjacent tetrahedra of equal size. Here *K* is the vibrational elastic constant and Δ_Os_ is the amplitude of the Os tetrahedron breathing mode. We expect *T*_c_ ~ *I*, given that the measured X-ray diffraction intensities (Fig. 5b) depend on these lattice distortions as *I* *~* *|f*_Os_Δ_Os_ *+* *f*_Cd_Δ_Cd_*|*^2^, where *f*_Os/Cd_ are X-ray atomic form factors. Hence *T*_c_ increases two orders of magnitude in a short pressure interval: from approximately 5 K at 37 GPa to ~500 K at 41 GPa, leading to the striking concave high-pressure phase boundary in Fig. 1.

## Discussion

The reduction of lattice symmetry from *Fd-3m* to *F-43m* in Cd_2_Os_2_O_7_ has direct implications for phonon coupling^3^ to spin fluctuations at the quantum critical point. Although spin-phonon coupling is known to generate unequal bond lengths in spin-Peierls dimers and antiferromagnetic superlattices^20^, the breathing phonons are not fully softened in the AIAO phase and, at least in the static limit, AIAO order only induces an external magnetostriction with a homogenous expansion^22^. In the high-pressure phase, a breathing lattice could in principle still permit AIAO spin configurations on different sized tetrahedra, despite a loss of site inversion symmetry in the *F-43m* space group. Nevertheless, as no long-range commensurate antiferromagnetic order was observed experimentally (Fig. 4c), the magnetic ground state at high pressure is likely spin-disordered, as ferromagnetically interacting Ising moments along local <1,1,1> axes would generate a high level of frustration^7^. Tuning by either chemical doping or pressure drives the ratio of the magnetic interaction strength to the hopping integral smaller in *A*_2_(Os,Ir)_2_O_7_. With an increasing electron density under 15% volume reduction by pressure, and moving away from the strong interaction strength limit^13^, one would not naturally expect a ferromagnetic ground state. Furthermore, ferromagnetic quantum phase transitions, as well as commensurate-incommensurate antiferromagnetic transitions, are first order, which would contrast with the continuous AIAO quantum phase transition observed in our experiment.

The increased bandwidth under pressure suggests that the electronic properties of Cd_2_Os_2_O_7_ in the high-pressure *F-43m* state become more metallic, potentially even superconducting in analogy to superconducting Cd_2_Re_2_O_7_ with its broken inversion symmetry (ref.^23^). The relationship between the spin and charge transitions is also intriguing. AIAO magnetic order and the metal-insulator transition respond similarly to compression across the *P–T* phase diagram. Our projected magnetic phase boundary in Fig. 1 gives d*T*_N_/d*P* ~ −5.0 K GPa^−1^ at *P* = 0, which is consistent with d*T*_MIT_/d*P* *=* −4 K GPa^−1^ in Cd_2_Os_2_O_7_ measured over the first 2 GPa range^14^. Comparing the two 5*d* AIAO ordered compounds with clear high-temperature metallic states, we find an average d*T*_N_/d*P* ~ −6.5 K GPa^−1^ over the whole AIAO phase in Cd_2_Os_2_O_7_ and a d*T*_MIT_/d*P* ~ −5.8 K GPa^−1^ in Nd_2_Ir_2_O_7_^17,18^. This comparison holds true despite large differences in *T*_N_ of 227 K and 33.5 K, respectively.

The experimentally observed coincidence of magnetic and structural phase transitions at *P*_c_ = 35.8 GPa, along with the similarities in the pressure evolution of the spin and charge degrees of freedom, point to one critical point within our experimental resolution (~1 GPa in the region around *P*_c_) between the insulating AIAO order and the spin-disordered metallic phase. As Landau’s phase transition theory would dictate two quantum phase transitions to be separated or to share a first-order phase line. If the transitions are separated, then our results point to a narrow intermediate region where the various spin, charge, and structural modes can couple across phases. If the phase boundaries actually meet at one critical point, then we may be in the realm of deconfined quantum criticality^24^. In any case, the concurrence of magnetic and structural phase transitions within our experimental resolution, and the clear deviation from mean-field behavior of the high-pressure phase, stimulate a discussion of strong coupling in the quantum critical region.

From the band structure perspective, the Os 5*d t*_2g_ band in Cd_2_Os_2_O_7_ is neither degenerate and forming a *S* = 3/2 state under Hund’s rule, as indicated by the reduced staggered moment <*m*> = 0.59 μ_B_ Os^−1^, nor cleanly separated into several narrow bands as demonstrated for a perfect OsO_6_ octahedron^14,25,26^. Instead, the *t*_2g_ orbitals in Cd_2_Os_2_O_7_ extend continuously over a spectral width of order 2 eV from the combined effect of *U* (~1 eV)^27^, spin-orbit coupling (~0.35 eV)^26^, and trigonal distortion (~0.3 eV) on the OsO_6_ octahedron^19^. Through the continuous quantum phase transition, the overall stability of the empty *t*_2g_ band is verified by the constant charge resonance profile at (4, 2, 0), with a coarse energy resolution slightly above 1 eV. From a metallic paramagnetic state, the formation of antiferromagnetic order would influence the oscillating dynamic component of the quasiparticle self-energy^28^, and in turn introduce a spin-order dependent repulsion between empty and filled states that could account for the insulating state below *P*_c_. This effective exchange field due to neighboring opposite spins is a generalized Slater mechanism, even without introducing Brillouin zone folding^28^. If the gap opens through a critical state of singular points at the Fermi surface, instead of a removal of states altogether, its thermodynamics could fit the Lifshitz description^27,29^.

The essentially concurrent, continuous quantum phase transitions of antiferromagnetism, structure, and (apparently) charge in Cd_2_Os_2_O_7_ provides the necessary ingredients for a generic approach to strongly-coupled, non-mean-field quantum criticality in three-dimensions^5^. With the Fermi surface fully gapped, quasiparticle fluctuations would involve all itinerant states in reducing the screening on Coulomb *U*, and the increased interaction range would then help stabilize a continuous quantum phase transition^5^. Indeed, at the ambient-pressure metal-insulator transition in Cd_2_Os_2_O_7_, an increase in *U* from 0.8 to 1.5 eV in the theoretical modeling is consistent with the observed spectral weight shift in infrared conductivity over the broad range of 0–4 eV (ref.^30^). Above the quantum critical point and in the spin-disordered phase space of the *Fd-3m* space group, soft AIAO spin fluctuations and lattice breathing modes could exist and compete, and further couple to quasiparticle fluctuations. The competition between spin and lattice degrees of freedom might explain the remarkable concave-shaped phase line at high pressure, as *T*_c_ scales to pressure with a non-trivial exponent much larger than one, a characteristic of strongly-coupled quantum criticality^5^. We note as well that the quantum critical region is asymmetric in *P–T* phase space, as the magnetic and structural phase lines approach *P*_c_ with different asymptotic behavior.

The quantum phase transition in Cd_2_Os_2_O_7_, with its interwoven spin, orbit, lattice, and charge degrees of freedom, contrasts sharply with systems that have a partially gapped Fermi surface, exemplified by itinerant spin density waves where persistent carriers screen spin fluctuations and lead to mean-field behavior^4–6,31^. The cubic AIAO antiferromagnet also differs from itinerant ferromagnets, where strong spin and charge mode coupling at wave vector **q** = 0 categorically induces first-order quantum phase transitions^4^. Spin-orbit coupling in 5*d* systems is regarded as intermediately strong^13^, and pressure drives *U*/*t* smaller with increasing kinetic energy *t*, away from the strong-correlation limit. Pressure tuning thus likely induces a continuous quantum phase transition while still preserving non-trivial quantum criticality in this 5*d* antiferromagnet. By Luttinger’s theorem, a continuous insulator-metal transition would result in either a carrier-mass enhancement or non-Fermi liquid behavior^32^ in Cd_2_Os_2_O_7_’s high-pressure phase, and the broken inversion symmetry sets the conditions for odd-parity superconductivity. A microscopic theory remains to be developed to describe the interaction between the AIAO spin fluctuations, breathing phonon modes, and quasiparticle excitations, especially taking into consideration the symmetry, chirality, and wave vector characteristics of each.

## Methods

### Resonant X-ray diffraction under high-pressure

Both charge orbital order and magnetic order can induce resonant behavior at an absorption edge in X-ray diffraction^9,12,31^. At the Os *L*_2_ edge, these two types of resonances share the empty part of the *t*_2g_ band as the intermediate state, and exhibit the same resonance profiles. The charge resonance originates from the anisotropic tensor susceptibility. In general, it can be observed at many forbidden lattice orders and, specifically, at both (4, 2, 0) and (6, 0, 0) in Cd_2_Os_2_O_7_. To avoid the charge anisotropic tensor susceptibility resonance and to isolate the magnetic resonance of the AIAO order, X-ray diffraction of the (6, 0, 0) order in Cd_2_Os_2_O_7_ was performed with a limited azimuthal angle range around 45° relative to the (0, 0, 1) vector, using a horizontal diffraction geometry for both *π*–σ and *π–π*’ polarization analyses^9^.

For the diamond anvil cell high-pressure environment^33^, we used a transmission (Laue) diffraction geometry in contrast to the typical reflection (Bragg) geometry at ambient pressure^9^. To achieve the diffraction geometry with the specified azimuthal condition at (6, 0, 0), single crystal samples were prepared in thin plate form with a surface normal along (0, −1, 1). This allows access to other diffraction orders such as (1, 1, 1), (4, 0, 0), (0, 2, 2), and (4, 2, 0) within the confined diamond anvil cell geometry, with the forbidden order (4, 2, 0) providing access to the charge-based anisotropic tensor susceptibility resonance. Several plates were polished down to 13 μm thickness, equivalent to one absorption length in Cd_2_Os_2_O_7_ for X-rays at the Os *L*_2_ edge (*E* = 12.387 keV). Unlike the iridates, resonance behavior at both the *L*_2_ and *L*_3_ edges are present in osmates. We chose the resonance at the *L*_2_ edge at high *P* because the higher energy X-rays had a longer X-ray penetration length through both the pressure environment and the sample.

We used the low-temperature, high-pressure diffraction setup at beamline 4-ID-D of the Advanced Photon Source^33^. To reduce absorption and enhance the signal-to-background ratio, a pair of wide-angle perforated Boehler diamond anvils^33^ (SYNTEK Co. LTD., Japan) were used, with culet size varying from 800 to 550 μm. A methanol/ethanol 4:1 mixture was used as the pressure medium inside rhenium gaskets. Pressure was calibrated by a Ag manometer in situ at 4.0 ± 0.5 K using a two-parameter Birch equation of state, with *B*_0_ = 108.85 GPa and *B*’ = d*B*/d*P* = 5.7 over the large pressure range. For X-ray polarization analysis, a highly oriented pyrolytic graphite (HOPG) plate of 5 mm thickness and 0.35° FWHM mosaic was used as the polarization analyzer. The (0, 0, 10) diffraction order of graphite at the Os *L*_2_ edge of 12.387 keV introduces a leakage of approximately 1.3% of the intensity from the *π–π*’ channel to the *π*–σ channel, and vice versa. Data presented here were collected from a total of 8 samples under pressure for spin (7) and charge (4) resonances. The absence of high-pressure commensurate antiferromagnetic order was verified on two crystals.

### Optical Raman scattering

Shards of single crystal Cd_2_Os_2_O_7_ with original growth surfaces were individually loaded with a Neon pressure medium in a diamond anvil cell, and subsequently thermally cycled in a liquid helium flow cryostat. Optical Raman scattering was performed using a Horiba LabRam HR Evolution system in the MRSEC facilities at the University of Chicago, equipped with a 633 nm wavelength laser for excitation.

### Data availability

The data that support the findings of this study are available from the corresponding authors upon request.
