# Publisher Correction: Strongly-coupled quantum critical point in an all-in-all-out antiferromagnet

**DOI:** 10.1038/s41467-018-05881-3

**Published:** 2018-09-05

**Authors:** Yishu Wang, T. F. Rosenbaum, A. Palmer, Y. Ren, J.-W. Kim, D. Mandrus, Yejun Feng

**Affiliations:** 1 Division of Physics, Mathematics, and Astronomy, California Institute of Technology, Pasadena, CA 91125 USA; 2 Department of Physics, The James Franck Institute, The University of Chicago, Chicago, IL 60637 USA; 3 The Advanced Photon Source, Argonne National Laboratory, Argonne, IL 60439 USA; 4 Department of Materials Science and Engineering, University of Tennessee, Knoxville, TN 37996 USA; 5 Materials Science and Technology Division, Oak Ridge National Laboratory, Oak Ridge, TN 37831 USA; 6 Okinawa Institute of Science and Technology Graduate University, Onna, Okinawa 904-0495 Japan

Correction to: *Nature Communications* 10.1038/s41467-018-05435-7; published online 27 July 2018

The original PDF version of the Article contained an error in the last sentence of the author affiliation information, which incorrectly read ‘Correspondence and requests for materials should be addressed to T.$.R. (e-mail: tfr@caltech.edu) or to Y.F. (e-mail: yejun@oist.jp)’. The correct version states ‘T.F.R.’ in place of “T.$.R.’. This has been corrected in the PDF version of the Article. The HTML version was correct from the time of publication.

